# SAR Image Simulations of Ocean Scenes Based on the Improved Facet TSM

**DOI:** 10.3390/s23052564

**Published:** 2023-02-25

**Authors:** Tong Wang, Ximin Li, Yijin Wang

**Affiliations:** 1 Air Defense and Antimissile School, Air Force Engineering University, Xi’an 710051, China; 2 National Laboratory of Radar Signal Processing, Xidian University, Xi’an 710071, China

**Keywords:** facet-based two scale model (FTSM), facet size, cutoff parameter, synthetic aperture radar (SAR)

## Abstract

The facet-based two scale model (FTSM) is widely applied in SAR image simulations of the anisotropic ocean surface. However, this model is sensitive to the cutoff parameter and facet size, and the choice of these two parameters is arbitrary. We propose to make an approximation of the cutoff invariant two scale model (CITSM) to improve the simulation efficiency while remaining the robustness to cutoff wavenumbers. Meanwhile, the robustness to facet sizes is obtained by correcting the geometrical optics (GO) solution, taking into account the slope probability density function (PDF) correction induced by the spectrum within an individual facet. The new FTSM, with less dependence on cutoff parameters and facet sizes, is proved to be reasonable in the comparisons with advanced analytical models and experimental data. Finally, SAR images of the ocean surface and ship wakes with various facet sizes are provided to prove the operability and applicability of our model.

## 1. Introduction

Microwave remote sensing of the marine environment has been an active research area since the advent of synthetic aperture radar (SAR) technology. Image simulations perform an essential role in the study of SAR technology. On the one hand, simulating SAR images under various parameters is of great help to understand and analyze different scattering mechanisms of the ocean surface. On the other hand, such simulations enable us to interpret real SAR images more accurately, making it easier to detect and identify various targets [1].

As the basis of SAR image simulations, investigations of the electromagnetic scattering models for ocean scenes are paramount. The scattering models can be separated into deterministic and statistical models depending on whether a deterministic sea surface is required. The statistical models, such as the small perturbation method (SPM) [2], Kirchhoff approximation (KA) [3], small slope approximation (SSA) [4], and integral equation method (IEM) [5], are simple in form, clear in physical interpretation, and are efficient in the calculation. However, rather than the local characterizations required for SAR imaging, statistical models can only offer the average scattering cross section from rough sea surfaces. The deterministic models include numerical models (method of moment [6], and time domain finite difference [7]), and approximate models (physical optics [8], and ray tracing method [9]). Although the numerical models are very accurate, the extremely high computing burden makes them impractical for huge ocean scenarios. The approximate models make some accuracy sacrifices in favor of greater computing efficiency. These deterministic models need to discretize the sea surfaces finely, typically with elements no larger than λ/8 (where λ is the wavelength of the incident waves). According to the most recent research, the surface element size must be as small as λ/478 in the C band to adequately account for the scattering contributions from all scales [10]. Even when employing the conventional discrete principles, dealing with large sea scenes results in a huge number of unknowns that must be resolved, severely limiting the effectiveness of SAR image simulations. For this problem, a semi-deterministic method has been developed to simulate Doppler signals and SAR images in the marine environment. Within its framework, the sea surface is modeled by a series of several slanted faces, and statistical models are used to determine the scattering strength of each facet. Within its framework, the sea surface is approximated by a succession of many tilted facet, and statistical models are used to determine the scattering strength of each facet. To determine the local scattering characteristics of maritime scenes for SAR imaging at the intermediate incidence angle, Franceschetti proposed a facet scattering model based on the KA solution [11,12]. Arnold-Bos and Khenchaf extended the classical TSM to a facet form for simulating SAR images of ship wakes in the bistatic configuration [13,14]. Chen improved the facet-based TSM (FTSM) by adding GO solution to the normalized radar cross section (NRCS) of individual facet [15], and Wu adopted this model to investigate the scattering field of the multi-scale deterministic sea surface with internal waves [16]. Differing from the above models that merely consider scattering amplitudes, Zhang developed the capillary wave modified facet scattering model (CWMFSM) [17], and an improved form of this model was presented in the subsequent research for SAR imaging of sea spikes [18]. Wei and Guo developed a sea spike scattering model based on the modified FTSM, which is similar to Zhang’s model [19]. Linghu increased the efficiency of the FTSM with GPU technology [20]. Recent attempts have been made by certain academics to deal with the contributions of Bragg scattering using models other than SPM [21]. Li introduced a facet scattering model developed from SSA1, and allowed for the use of large facet to evaluating the sea scattering field [22]. More recently, the scattering issue of sea surfaces covered with crest foam and static foam was addressed using a modified facet scattering model, in which IEM was employed to analyze the small-scale contribution instead of SPM [23]. It should be noted that all the above semi-deterministic models may be seen as FTSM or its deformed form, and their implementations must address two issues. The first is the selection of cutoff wavenumbers used to divide the sea spectrum into large and small parts. In classical TSM, the choice of cutoff parameter is arbitrary, and the varying values of this parameter significantly impact the computed results. In order to build a more tractable TSM, Soriano proposed the cutoff invariant TSM (CITSM) [24], in which SPM was replaced with the first order small slope approximation (SSA1), and the specular scattering was corrected. However, as compared to the conventional TSM, the integral process of SSA1 significantly reduces computing efficiency [25]. The other issue is the specification of facet sizes. The exact selection principle is not covered in the majority of the published studies, despite the fact that facet sizes in semi-deterministic models are permitted to be substantially larger than the incident wavelength. In our earlier research [26], we performed a preliminary analysis of the facet scattering model based on CITSM, but did not conduct in-depth analyses of SAR image simulations of anisotropic sea surfaces. Considering the current development of facet TSM and the requirements of SAR imaging, we aim to develop an effective and broadly applicable FTSM with little dependency on the cutoff parameter and facet size.

In this paper, we first derive the approximated form of CITSM in Section 2. In Section 3, the FTSM based on simplified CITSM and modified slope probability density function (MSPDF) is given. Section 4 illustrates applications of the new FTSM in the SAR imaging of ocean surfaces.

## 2. Simplified Form of Cutoff Invariant TSM

**Figure 1:**
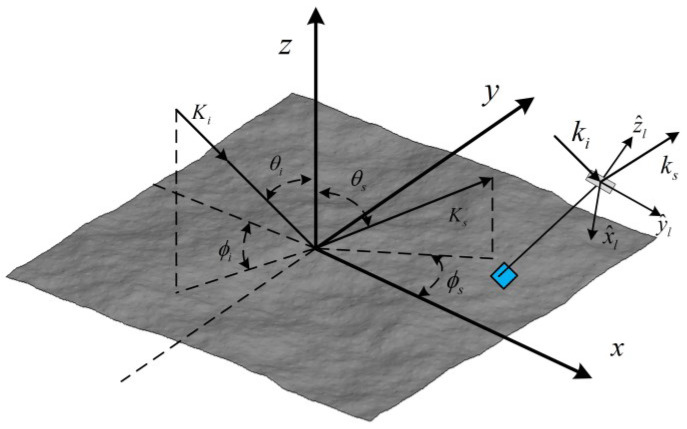
Geometric configuration of scattering from ocean surface in two scale profile.

First, we recall the geometrical configuration of the scattering problem of ocean surfaces. As shown in Figure 1, the global frame of reference system is defined as $\left(\hat{x},\hat{y},\hat{z}\right)$, and $K_{i}$ and $K_{s}$ denote incident and scattered wave vector, respectively, $Q=K_{s}-K_{i}$, $\theta_{i}$, and $\theta_{s}$ are the incident and scattered angles, $\varphi_{i}$ and $\varphi_{s}$ are the azimuth incident and scattered angles. The sea surface elevation is described by h(r)=h(x,y), where r is the *x-y* plane component of the position vector R=(r, z). Similarly, The local facet is described by $h_{s}\left(r_{l}\right)=h_{s}\left(x_{l},y_{l}\right)$, and vector $r_{l}$ denote the in-plane component of the position with respect to the local frame of reference system $\left({\hat{x}}_{l},{\hat{y}}_{l},{\hat{z}}_{l}\right)$, $\hat{n}$ is the unite normal vector of the local facet, and $R_{0}$ is the position vector of its center. Within the local framework, $k_{i}=\left(k_{0},-q_{0}\right)$ and $k_{s}=\left(k,q_{k}\right)$ are the incident and scattered wave vectors, the vector $k_{0}$ and k are the *x_l_*-*y_l_* components, and $q_{0}$ and $q_{k}$ are the values of corresponding *z_l_* components.

According to TSM, two basic mechanisms that contribute to ocean scattering: the specular reflection caused by gravity waves, and the diffuse scattering caused by capillary waves modulated by the slope of large-scale roughness. The most commonly used expression of TSM is symbolically written as:

(1) $$\sigma =\mathrm{GO}+{\langle \mathrm{SPM}1\rangle }_{L}$$

where σ is the normalized radar cross section (NRCS), GO represents the specular reflection contribution, and SPM1 is the first order small perturbation method. ${〈\cdot 〉}_{L}$ denotes ensemble average over large scales. Based on (1), a scale-dividing parameter *k_c_* needs to be introduced to separate large and small scales of the ocean spectrum. However, the selection of *k_c_* lacks physical rationale, and this parameter is adopted more empirically.

### 2.1. Cutoff Invariant TSM 

In order to decrease the impact of cutoff wavenumber on the scattering power, a new model called CITSM was developed in [24], in which SPM1 is replaced with SSA1 for the evaluation of small-scale contributions. The NRCS in CITSM is expressed as:

(2) $$\begin{array}{c} \sigma \left(K_{s},K_{i}\right)=\sigma_{\mathrm{GO}}\times \exp\left(-Q^{2}h_{S}^{2}\right)\left(1-\exp\left(-Q_{z}^{2}h_{L}^{2}\right)\right)\\ +{\langle \sigma_{\mathrm{SSA}1}\left(k_{s},k_{i}\right)\rangle }_{L} \end{array}$$

where $h_{S}$ and $h_{L}$ are the root mean squire heights (RMSH) of small-scale and large-scale parts, respectively. *Q* and *Q*_z_ are the norm and vertical component of vector ***Q***. $\sigma_{\mathrm{GO}}$ is the classical GO solution, and $\sigma_{\mathrm{SSA}1}\left(k_{s},k_{i}\right)$ denotes the incoherent NRCS of the local facet obtained by SSA1. Analyzing formula (2), we find that when the cutoff wavenumber *k_c_* moves toward a higher value, the value of $h_{S}$ becomes smaller, and $h_{L}$ becomes larger, resulting in an augmentation of the first term on the right side in (2). Meanwhile, the second term corresponding to the SSA1 solution gets smaller due to the decrease in small-scale components. As a result of mutual compensation between these two terms, the total NRCS remains almost constant, thus obtaining the robustness to cutoff wavenumbers. 

The incoherent NRCS by SSA1 solution in the local facet frame can be expressed as follows:

(3) $$\begin{array}{l} \sigma_{\mathrm{SSA}1}=\frac{1}{\pi }{\left|\frac{2q_{k}q_{0}}{q_{k}+q_{0}}B_{pq}\left(k,k_{0}\right)\right|}^{2}\exp\left[-{\left(q_{k}+q_{0}\right)}^{2}C\left(0\right)\right]\\ \times \int_{S}\left\{\exp\left[{\left(q_{k}+q_{0}\right)}^{2}C\left(r\right)\right]-1\right\}\exp\left[-i\left(k-k_{0}\right)\cdot r_{l}\right]dr_{l} \end{array}$$

where $B\left(k,k_{0}\right)$ is the SPM kernels, and its explicit expression can be referred to [27]. *p* and *q* denote the polarizations of scattered and incident radar waves, and *C*(***r***) is the autocorrelation function of the ocean surface that defined as:

(4) $$C\left(r\right)=\int_{0}^{2\pi }d\varphi \int_{0}^{\infty }W\left(K\right)\exp\left(iK\cdot r\right)dK$$

where *W*(***K***) is the directional ocean spectrum, and ${C(0)=h}^{2}$ represents RMSH of the whole spectrum within an individual facet. For the Elfouhaily ocean spectrum [28], it is convenient to simplify the integration process in (4) into an analytical solution with the help of Bessel functions. The autocorrelation function of the Elfouhaily sea surface can be written as:

(5) $$C\left(r\right)=C\left(r,\phi \right)=C_{0}\left(r\right)-\cos\left(2\phi \right)\times C_{2}\left(r\right)$$

Additionally, the calculations of $C_{0}\left(r\right)$ and $C_{2}\left(r\right)$ are performed as:

(6) $$\begin{array}{l} C_{0}\left(r\right)=\int_{0}^{\infty }W\left(K\right)J_{0}\left(Kr\right)dK\\ C_{2}\left(r\right)=\int_{0}^{\infty }W\left(K\right)J_{2}\left(Kr\right)\Delta \left(K\right)dK \end{array}$$

where $J_{0}$ and $J_{2}$ are zero and second order Bessel functions of the first kind.

### 2.2. Simplification of CITSM

Due to the integrated process of the SSA1 solution, CITSM takes substantially longer than the conventional TSM (GO-SPM). Especially for the anisotropic ocean surfaces, no one closed-form solution can be derived from SSA1. Although the two-dimensional integral of the *C*(***r***) evaluation in Formula (4) can be simplified into two one-dimensional integrals, as shown in Formula (6), the computational burden is also severe. Note that the SSA1 is integrated only over small-scale components of the ocean surface in CITSM, i.e., the integral domain of *C*(***r***) in (4) is $\left[k_{c},\;\infty \right]$. From this, we expect to derive a reasonable and tractable expression with high calculated efficiency. For the exponential factor in Formula (3), if *C*(***r***) is small enough, then the following condition is fulfilled.

(7) $${\left(q_{k}+q_{0}\right)}^{2}C\left(r\right)\ll 1$$

Additionally, the exponential factor can be approximated by:

(8) $$\exp\left[{\left(q_{k}+q_{0}\right)}^{2}C\left(r\right)\right]\approx 1+{\left(q_{k}+q_{0}\right)}^{2}C\left(r\right)$$

Insertion of this asymptotic approximation in SSA1 integral in Formula (2) leads to the approximated solution.

(9) $$\begin{array}{c} \sigma_{\mathrm{SSA}1}=\frac{1}{\pi }{\left|2q_{k}q_{0}B_{pq}\left(k,k_{0}\right)\right|}^{2}\exp\left[-{\left(q_{k}+q_{0}\right)}^{2}C\left(0\right)\right]\\ \times \int_{S}C\left(r\right)\exp\left[-i\left(k-k_{0}\right)\cdot r_{l}\right]dr_{l} \end{array}$$

For a stationary random process, as we know, the spectrum distribution and its autocorrelation function have the following relationship:

(10) $$W\left(K\right)=\frac{1}{4\pi^{2}}\int C\left(r\right)\exp\left(-iK\cdot r\right)dr$$

Now, substituting (10) into (9), then the new solution is given by:

(11) $$\begin{array}{l} \sigma_{\mathrm{SSA}1}\left(k,k_{0}\right)=16\pi q_{k}q_{0}{\left|B_{pq}\left(k,k_{0}\right)\right|}^{2}\exp\left[-{\left(q_{k}+q_{0}\right)}^{2}h_{s}^{2}\right]\\ \;\;\;\;\;\;\;\;\;\;\;\;\;\;\;\;\;\;\;\;\;\;\;\;\;\;\times W_{S}\left(k-k_{0}\right)\\ \;\;\;\;\;\;\;\;\;\;\;\;\;\;\;\;\;\;\;\;\;\;\;\;\;\;=\sigma_{\mathrm{SPM}}\exp\left[-{\left(q_{k}+q_{0}\right)}^{2}h_{s}^{2}\right] \end{array}$$

where $W_{S}$ denotes small-scale components of the ocean spectrum, and $\sigma_{\mathrm{SPM}}$ represents SPM1 solution. Recalling (2) and the analytical solution of CITSM is rewritten as:

(12) $$\begin{array}{c} \sigma \left(K_{s},K_{i}\right)=\sigma_{\mathrm{GO}}\times \exp\left(-Q^{2}\sigma_{S}^{2}\right)\left(1-\exp\left(-Q_{z}^{2}h_{L}^{2}\right)\right)\\ +{\langle \sigma_{\mathrm{SPM}}\exp\left[-{\left(q_{k}+q_{0}\right)}^{2}h_{s}^{2}\right]\rangle }_{L} \end{array}$$

We explicitly note the exponential quantity in the second term of (12) acts as an attenuation factor, and its impact on calculated results gets more significant when the incident vector approaches the nadir. The impact can be neglected if the waves incident at near grazing angles.

**Figure 2:**
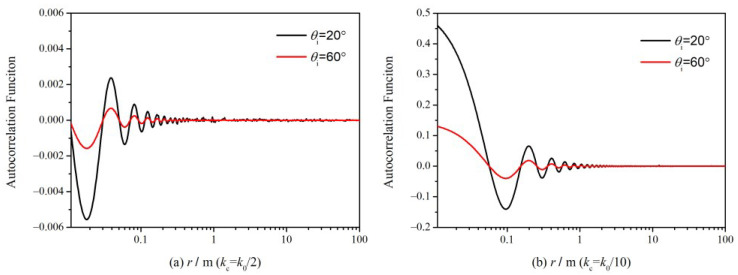
*I* versus distance simulated with different cutoff wavenumbers and incident angles.

Only when (7) is fulfilled, the analytical solution in (12) is available. Hence, we need to illustrate how the autocorrelation coefficient changes with cutoff parameters. For simplicity, we set:

(13) $$I\left(r\right)=4k_{0}^{2}\cos^{2}\theta_{i}C\left(r,0\right)$$

where *C*(*r*, 0) corresponds to (5) in which ϕ is set to zero, and *I*(*r*) denotes the value of term ${\left(q_{k}+q_{0}\right)}^{2}C\left(r\right)$ in the backscattering case. The frequency of incident waves is 14 GHz, and the ocean surface is generated via the Elfouhaily spectrum (a detailed description will be presented in Section 3), and the wind speed is 5 m/s. Figure 2 shows the variation of I as a function of distance *r* under different incident angles and cutoff wavenumbers.

As shown in Figure 2, *I* is always far smaller than 1, especially for the cases with large incident angles, which indicates that the formula in (7) may be applied to a wide range of cutoff wavenumbers and wind speeds. As a result, the evaluation of ocean NRCS can use the analytical solution in (12). It must be noted that the cutoff wavenumber chosen cannot be too small to avoid errors in the nadir region. Nevertheless, it is also a robust model over a broad domain of cutoff wavenumbers. 

**Figure 3:**
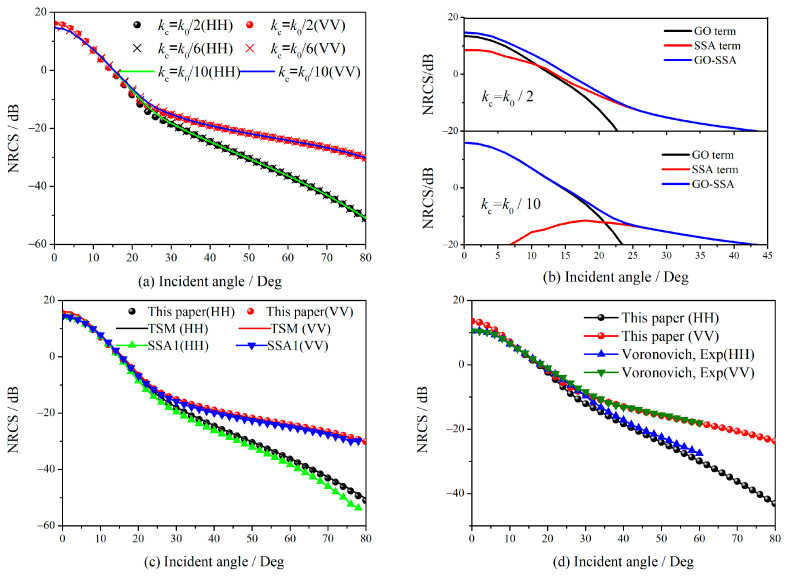
Comparison of backscattering NRCS using simplified form in formula (12), the frequency of incident waves is 14 GHz, wind speed in (**a**–**c**) is 5 m/s, in (**d**) is 10 m/s.

Figure 3 shows NRCS of the ocean surface obtained by (12) at various cutoff wavenumbers. Along with the results calculated by SSA1 and TSM, the experimental data are shown to illustrate the accuracy. The excellent agreement for NRCS at various cutoff wavenumbers shown in Figure 3a illustrates how little the simplified CITSM depends on cutoff wavenumbers. Figure 3b illustrates the behavior of the first (GO term) and second (SSA term) terms in (12) for the cutoff parameters *k*_0_/2 and *k*_0_/10. These terms account for the specular and diffuse scattering contributions, respectively. It demonstrates that as the cutoff wavenumber changes, the specular and diffuse terms also change. However, the NRCS of CITSM are independent of cutoff wavenumbers because the decrease in one component is balanced out by the increase in the other. The comparisons in Figure 3c illustrate that the results predicted by the simplified CITSM are in good agreement with SSA1 and TSM. In Figure 3d, the data from the experiment of Voronovich and Zavorotny [27] is also offered to validate the accuracy of simplified CITSM. The performance of the simplified CITSM in Figure 3 exemplifies its benefit of obtaining the same accuracy as TSM and SSA1 with little dependency on cutoff wavenumbers. There is no integral process in the simplified form in (12), which is much more efficient than the original form in (2). On the same computer, for instance, simulating the NRCS takes 352 s with the original CITSM, but just 23 s with the simplified CITSM. The significant improvement in simulation efficiency enables the simplified CITSM to be applied in the scattering evaluation of large sea surface as a facet form. 

## 3. Facet-based Scattering Model

### 3.1. Discussion of the Facet Size 

In contrast to statistical scattering problems, deterministic scattering scenarios need the creation of a surface geometric shape. The height map of ocean surfaces can be generated by using a 2D Fourier extension with the help of a given ocean spectrum. The number of discrete elements depends on two parameters, i.e., the length *L* and sampling interval Δx, and these two parameters can be related to the wavenumber domain by using the following relations:

(14) $$K_{L}=\frac{2\pi }{L}\;\;\;\;\;\;K_{S}=\frac{\pi }{\Delta x}$$

The length *L* and sampling interval Δx must be carefully chosen before simulations of the scattering power are performed, as too long a length or too short an interval will result in a significant computational burden, while an excessively small length or large interval will produce nonphysical results. Therefore, the length *L* and sampling interval Δx specifications are essential for developing a facet-based scattering model.

The real wave spectrum described by the discrete ocean surface is a truncated result. Based on (14), *L* and Δx are determined by *K*_L_ and *K*_S_ corresponding to the low and high truncated wavenumber, respectively. The spectrum components below *K*_L_ or above *K*_S_ cannot be included in the deterministic ocean surface. In other words, a good representation of marine geometric features depends on the choice of the length and sampling interval. According to the conclusions in [10], for a realization of the ocean surface with 5 m/s wind speed, the length is at least 50 m long corresponding to 1667 λ at the X band or 2333 λ at the Ku band. If the wind speed increases to 10 m/s, the length reaches 135 m long (4500 λ for the X band, and 6300 λ for the Ku band), which is electrically large for the scattering field evaluation. As for the sampling interval, Δx is required to be selected as fine as 0.0166 λ at the X band, and 0.0232 λ at the Ku band. Personal computers cannot handle such a dense discretization of the ocean surface scattering problem. The facet-based scattering model is suggested in a semi-deterministic manner to decrease the number of facets. In this scattering model, the rough surface is generated by the large-scale roughness, while the small-scale components can be represented in a statistical way. In terms of physical mechanics, this model is comparable to TSM, and supports large facet sizes thanks to the use of analytical methods to determine local scattering contributions. However, the selection of facet sizes is less theoretical and more empirical. For this reason, within the TSM scheme, we aim to develop a new facet-based scattering model that is robust to the variation of facet size and facilitates the calculation of ocean scattering using large facet elements.

### 3.2. Facet-Based TSM

Based on Facet-based TSM (FTSM), an ocean surface consisting of a series of facets is first modeled, and the geometric information imposed on every facet is described statistically. Then, the scattering cross section of the individual facet can be evaluated with respect to the local incident angle. Finally, the average scattering coefficient is obtained by summing the cross sections of all the facets and dividing the total area. The scattering contribution in FTSM is composed of specular and diffuse components, and is generally expressed by [15]:

(15) $$\sigma_{pq}=\frac{\nu \left(\theta_{i},\theta_{s}\right)}{A}\sum_{i=1}^{M}\sum_{j=1}^{N}{\left[\left(\sigma_{ij}^{\mathrm{GO}}+\sigma_{ij}^{\mathrm{SPM}}\right)\Delta x\Delta y\right]|}_{z_{xij}\in \left[\partial_{ij}\right]|\partial_{ij}>-\cot\theta_{i}}^{z_{yij}\in \left[\beta_{ij}\right]}$$

where *p* and *q* are the polarizations of scattered and incident waves, *v*(*θ_i_*, *θ_s_*) is the visibility factor, and *A* is the area of the illuminated sea surface. The area of each facet is Δ*x* × Δ*y*. *z_xij_* and *z_yij_* are slopes along *x* and *y* directions of the (*i*, *j*) facet. Owing to the shadowing effect, values of *z_x_* need to be larger than –cot*θ_i_*. In FTSM, a suitable cutoff parameter is also essential as TSM, which is able to smooth the transition region between the specular and diffuse components. Inspired by the simplified CITSM in Section 2, we applied this model to a facet form. Similar to formula (15), the facet form of CITSM is given as:

(16) $$\begin{array}{l} \sigma_{pq}=\frac{\nu \left(\theta_{i},\theta_{s}\right)}{A}\sum_{i=1}^{M}\sum_{j=1}^{N}[(\sigma_{ij}^{\mathrm{GO}}\exp\left(-q_{ij}^{2}h_{S}^{2}\right)\left(1-\exp\left(-q_{zij}^{2}h_{L}^{2}\right)\right)\\ {\;\;\;\;\;\;\;\;+\sigma_{ij}^{\mathrm{SSA}})]\Delta x\Delta y|}_{z_{xij}\in \left[\partial_{ij}\right]|\partial_{ij}>-\cot\theta_{i}}^{z_{yij}\in \left[\beta_{ij}\right]} \end{array}$$

where $\sigma_{ij}^{\mathrm{GO}}$ corresponds to the classical GO solution, $\sigma_{ij}^{\mathrm{SSA}}$ corresponds to the incoherent solution of SSA1, the denotations of the other parameters refer to the formulas (2) and (15). The diffuse term, i.e., $\sigma_{ij}^{\mathrm{SSA}}$ solution, is calculated by simplified CITSM as (11) to avoid tedious integral operations, thus making the implementation as efficient as traditional TSM. According the definition of the RMSH, for Elfouhaily ocean spectrum the $h_{S}^{2}$ and $h_{L}^{2}$ can be expressed as:

(17) $$h_{S}^{2}=\int_{k_{c}}^{\infty }W\left(K\right)dK,\;\;\;\;\;\;h_{L}^{2}=\int_{0}^{k_{c}}W\left(K\right)dK$$

where *W*(*K*) is the Elfouhaily omnidirectional spectrum, and *k*_c_ is the divided wavenumber. 

GO is the high-frequency limit of KA, and its validity domain is given for moderate angles by *kh* > π/2 (*h* is the RMSH of a rough surface). In the case of local facet scattering problems, as the RMSH within an individual facet is very small, the GO approximation becomes inaccurate. Hence, an improved GO model is needed to broaden the applicable domain to include facet-based scattering situations. In [29], Thompson introduced a more generalized GO. Based on this model, we rewrite the expression of GO as:

(18) $$\sigma_{pq}^{\mathrm{GO}}=\exp\left(-q^{2}h_{S}^{2}\right)\frac{\pi k_{i}^{2}q^{2}}{q_{z}^{4}}{\left|U_{pq}\right|}^{2}P\left(z_{x},z_{y}\right)$$

where *U_pq_* is the polarization factor and can be referred to [30]. Different from the simple Gaussian slope PDF used by Thompson, we adopt the Cox-Munk slope PDF, that is defined as:

(19) $$P\left(z_{x},z_{y}\right)=\frac{F\left(z_{x},z_{y}\right)}{2\pi \nu_{u}\nu_{c}}\exp\left(-\frac{z_{x}^{2}}{2\nu_{u}^{2}}-\frac{z_{y}^{2}}{2\nu_{c}^{2}}\right)$$

where *ν*_u_ and *ν*_c_ are the root mean square slopes (RMSS) in upwind, and crosswind directions, details of the *F*(*z_x_*, *z_y_*) are given in [31]. According to the Thompson model, the RMSS should be filtered by a cutoff wavenumber that separates the long waves and short waves (in this paper, we define this cutoff wavenumber as equivalent to *k*_c_ that is mentioned above). Thus, the *ν*_u_ and *ν*_c_ are redefined, respectively as:

(20) $$\nu_{c}^{2}=\int_{0}^{k_{c}}dk\int_{0}^{2\pi }{\left(k\sin\varphi \right)}^{2}S\left(k,\varphi \right)d\varphi$$

(21) $$\nu_{u}^{2}=\int_{0}^{k_{c}}dk\int_{0}^{2\pi }{\left(k\cos\varphi \right)}^{2}S\left(k,\varphi \right)d\varphi$$

By introducing the fileted RMSS, the GO solution in (20) can achieve the same accuracy as KA in the vicinity of the specular lobe, accounting for the fact that scale waves much shorter than radar wavelength are not “seen” by the radar [32]. As a result, we apply this improved GO in place of classical GO to evaluate the specular scattering of local facets.

**Figure 4:**
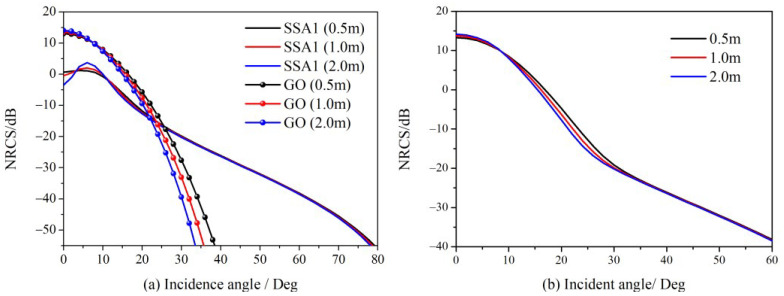
Backscattering NRCS of ocean surface with different facet size. (**a**) specular (GO term) and diffuse (SSA1 term) contributions; (**b**) total NRCS with different facet sizes.

The new FTSM is obtained by replacing the term $\sigma_{ij}^{\mathrm{GO}}\exp\left(-q^{2}h_{S}^{2}\right)$ in (16) with GO expression as (18), and based on this scattering model, NRCS for different facet sizes are simulated and shown in Figure 4. The wind speed is 5 m/s, the radar wave frequency is 14 GHz, and the relative dielectric constant of seawater is 42- j36. Figure 4a shows the curves of diffuse scattering contributions at incidence angles higher than 10 degrees, i.e., in the direction of non-specular reflection, which show relatively slight variation for different facet sizes. The facet size significantly affects the diffuse scattering for incidence angles smaller than 10 degrees, the specular scattering contribution dominates this region for the total NRCS. Namely, the specular scattering component of the overall scattering power mainly reflects the impact of facet size. The FTSM, which is robust to facet sizes, is possible if we correct the GO solution to lessen its dependence on facet sizes.

The GO curves in Figure 4a exhibit non-zero lobe behavior, with widths linked to the slope PDF *P*(*z_x_*, *z_y_*) of the whole sea surface. In fact, the slope PDF is no longer *P*(*z_x_*, *z_y_*) if an ocean surface is discretized into a sequence of facets, but rather merely a function of the partial sea spectrum within each facet. That is why the GO curves in Figure 4a vary with facet sizes. By adjusting the slope PDF of each facet, we are attempting to develop a novel GO solution that is independent of facet sizes.

The GO result for a deterministic scattering scenario is not contributed by small-scale roughness with wave numbers exceeding *k*_c_. The RMSS within a single facet is only affected by its own partial sea spectrum. Consequently, the integrals of new RMSS (named by *ν*_ul_ and *ν*_cl_) are only involved in the effective integration domain, e.g., $\left[k_{\mathrm{cl}},k_{c}\right]$, in which *k*_cl_ depends on the facet size. It leads to the following expressions:

(22) $$\nu_{\mathrm{cl}}^{2}=\int_{k_{\mathrm{cl}}}^{k_{c}}dk\int_{0}^{2\pi }{\left(k\sin\varphi \right)}^{2}S\left(k,\varphi \right)d\varphi$$

(23) $$\nu_{\mathrm{ul}}^{2}=\int_{k_{\mathrm{cl}}}^{k_{c}}dk\int_{0}^{2\pi }{\left(k\cos\varphi \right)}^{2}S\left(k,\varphi \right)d\varphi$$

where *k*_cl_ = 2π/Δ*x* (Δ*x* = Δ*y*). Replacing the *ν*_u_, *ν*_c_ in (19) with *ν*_ul_ and *ν*_cl_, respectively, the new slope PDF (defined as *P*_L_(*z_x_*, *z_y_*)) can be given, and the expression for specular scattering based on modified GO solution in the facet-based model is given as:

(24) $$\begin{array}{c} \sigma_{pq}^{\mathrm{MGO}}=\sum_{i=1}^{M}\sum_{j=1}^{N}\exp\left(-q_{ij}^{2}h_{S}^{2}\right)\left(1-\exp\left(-q_{zij}^{2}h_{L}^{2}\right)\right)\times \\ \frac{\pi k_{i}^{2}q_{ij}^{2}}{q_{ij}^{4}}{\left|U_{pq,ij}\right|}^{2}P_{L}\left(z_{x},z_{y}\right) \end{array}$$

**Figure 5:**
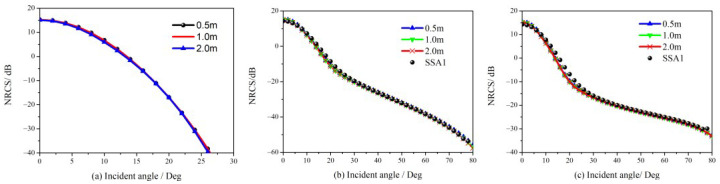
Comparison of NRCS simulated by the new model with different facet sizes. (**a**) NRCS of specular (GO term) contributions; (**b**) NRCS of total scattering contribution at HH polarization; (**c**) NRCS of total scattering contribution at VV polarization.

By substituting (24) into (16) for the expression of specular scattering, the new FTSM with robustness to facet sizes is obtained. NRCS of the ocean surface and its comparisons with different facet sizes are given in Figure 5, in which the conditions remain the same as in Figure 4. 

In Figure 5a, the new GO results perform much better than the classical model for different facet sizes, which demonstrates the new model is robust to the facet size. Figure 5b,c show a good agreement between the new model with different facet sizes and the SSA1 model. Figure 5 indicates that we can adopt flexible principles of facet sizes in dealing with the deterministic ocean scattering scenario to avoid a huge computational burden.

**Figure 6:**
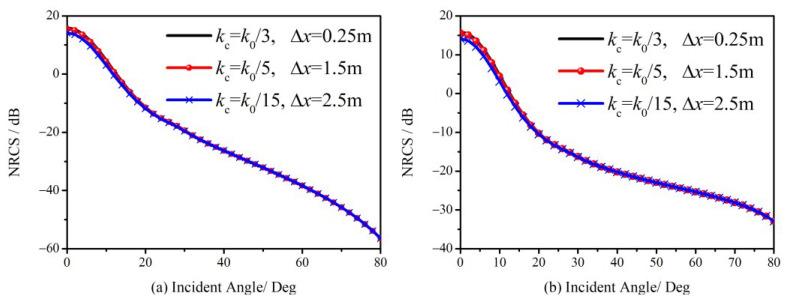
Comparison of NRCS simulated by the new model with different facet sizes and cutoff wavenumbers. (**a**) NRCS at HH polarization; (**b**) NRCS at VV polarization.

Figure 6 illustrates the simulated NRCS for different facet sizes and cutoff wave-numbers. Therefore, the proposed approach has the potential to make the scattering problem of the sea surface simpler. A simplified scattering model without sacrificing accuracy is appealing for radar imaging.

## 4. SAR Image Simulations of Ocean Scenes

Since the proposed FTSM is no longer sensitive to the facet sizes, as discussed before, we may use the large facet to decrease the computational cost without sacrificing accuracy. It is worth noting that we have focused mainly on textural characteristics rather than the pixel-level speckles that may be introduced to SAR images using statistical methods [33,34,35].

**Figure 7:**
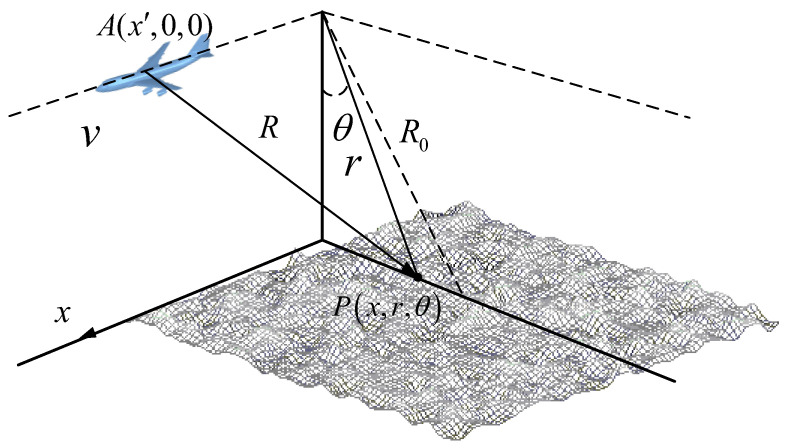
The geometry of ocean SAR.

Based on the geometry shown in Figure 7, SAR raw signal can be written as:

(25) $$\begin{array}{l} h_{\mathrm{strip}}\left(x^{\prime },r^{\prime }\right)=\iint \gamma \left(x,r\right)\exp\left(-j\frac{4\pi }{\lambda }\Delta R\right)\exp\left[-j\frac{4\pi }{\lambda }\frac{\Delta f}{fc\tau }{\left(r^{\prime }-r-\Delta R\right)}^{2}\right]\\ \cdot \omega^{2}\left(\frac{x^{\prime }-x}{X}\right)rect\left[\frac{2\left(r^{\prime }-r-\Delta R\right)}{c\tau }\right]dxdr \end{array}$$

where γ(y,r) is the reflectivity of a small facet located at (y,r) which can be obtained by (24). *R* is the path length between the radar sensor and the interest point P(y, r,θ). *R*_0_ is the minimum distance from the flight line to the origin of sea surface. Δf is the bandwidth of signal. *c* is light velocity, and τ is pulse duration. $X=\lambda R_{0}/L$ is the beam width at azimuth direction of the detected antenna. $\Delta R=R-r=\sqrt{r^{2}+{\left(y^{\prime }-y\right)}^{2}}$ and ω(·) are the antenna pattern. We simply defined that antenna gain is the same in the illuminated area. $r^{\prime }$ is the delay time of pulse multiply c∕2.

**Figure 8:**
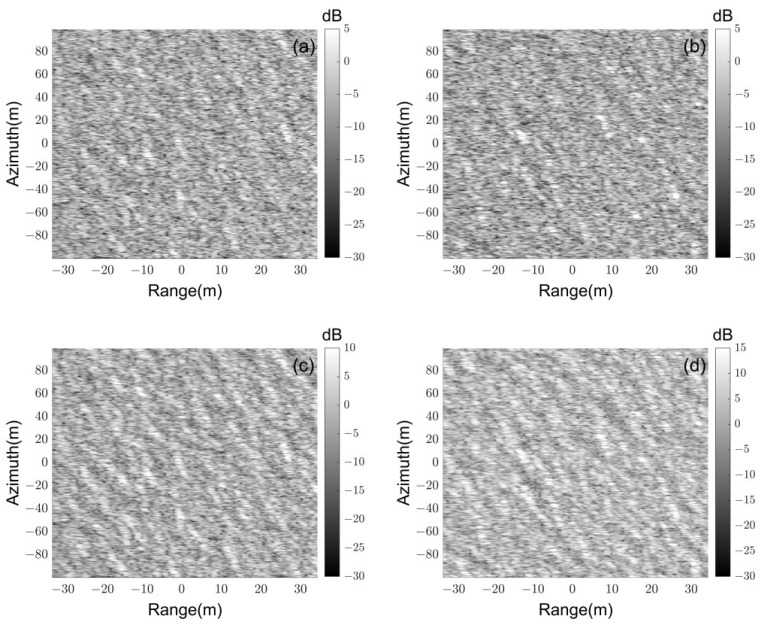
SAR images of ocean surface: RCS of each facet in image (**a**,**b**) are obtained with the new model, and (**c**,**d**) are obtained with the traditional facet–based TSM; the facet size in image (**a**,**c**) corresponds to 1 m, and (**b**,**d**) corresponds to 0.5 m.

With the help of (24), SAR images of the ocean surface simulated by the traditional and new FTSM with different facet sizes are given in Figure 8. The elevation map of the ocean surface is generated with the help of the Elfouhaily spectrum. The wind speed is 5 m/s, the direction is 45°, and the relative dielectric constant of seawater is 42- j36. The radar works at VV polarization, the resolutions along with the range and azimuth directions are 1.5 m, and the incident angle of radar waves is 20°. The other parameters can be found in Table 1.

**Figure 9:**
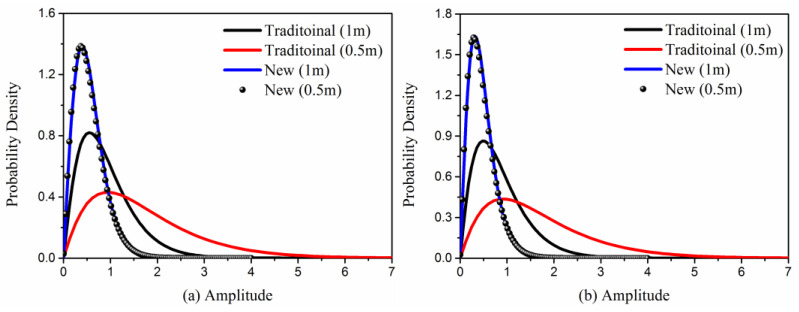
Amplitude histogram of SAR images simulated by traditional and new facet-based TSM: 1 m and 0.5 m mean the facet sizes in SAR image simulations, (**a**) at VV polarization;, and (**b**) at HH polarization.

Figure 8a,b show nearly comparable scattering power variation ranges, as would be predicted. However, Figure 8c,d show a notable difference. The statistical analysis of image amplitudes is done to demonstrate the impact of facet sizes on SAR images in more detail in Figure 9. It presents the amplitude histograms of SAR images simulated by the traditional and new models with different facet sizes. It shows that the amplitude distribution of SAR images generated with the facet size 1 m corresponds well to the facet size 0.5 m with the new FTSM.

By contrast, for classical FTSM, the variation in facet sizes causes a dramatic deviation in amplitude distributions of SAR images, accounting for its significant dependence on facet sizes. Recalling accuracy validations in Figure 5, it offers tremendous flexibility in selecting an acceptable facet size to increase simulation efficacy and can produce excellent SAR images with various facet sizes.

Ship wakes are one of the essential features in SAR pictures of the ocean scene because they indicate the changes in the ocean surface caused by ships. We utilize the new FTSM to simulate SAR images of ship wakes to demonstrate its applicability in representing local scattering characteristics.

**Figure 10:**
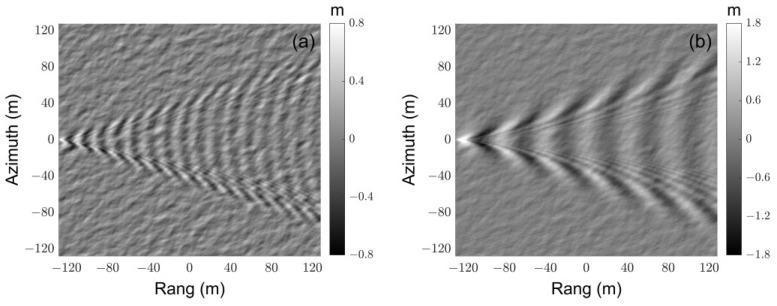

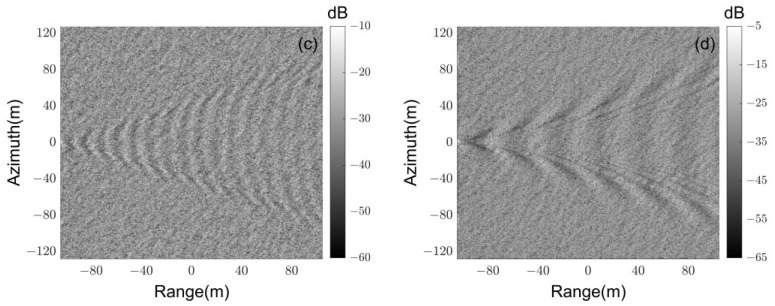
Height maps and SAR images of ocean-wake surface: (**a**,**b**) are height maps of ocean-wake surface, in (**a**) wind speed is 4 m/s and ship speed is 5 m/s, in (**b**) wind speed is 4 m/s and ship speed is 8 m/s; and (**c**,**d**) are SAR images corresponding to the (**a**,**b**), respectively.

The height maps and SAR images of ocean-wake surfaces are shown in Figure 10. 42- 30j. Ship wakes are modeled by the Kelvin wake [36], and the ship is 120 m in length, 20 m in width, and the wall-sided draft is 5 m. The dimension of the scene is 256 m × 256 m, and the facet size is set to 0.5 m. The radar works at HH polarization with strip-map mode, and the incident angle is 55°. Both the range and azimuth resolutions are 1 m. It can be seen that the features of wakes, as shown in Figure 10a,b, are well presented in the corresponding SAR images in Figure 10c,d. Ship wakes make the sea surface exceptionally rough, and considerably enhance diffuse scattering, and specular reflections dominate specific elementary contributions. Since ship wakes are known to continue for hours, these characteristics in SAR images are crucial for ship detection [37].

## 5. Conclusions

In this paper, we develop a new FTSM for SAR image simulations of the large and anisotropic ocean surface. The scattering contribution of each facet is obtained by means of the simplified CITSM, which is highly efficient in the calculation and is independent of the cutoff parameters. Additionally, by correcting the specular expression, the dependence on facet sizes is diminished, allowing for the use of fewer facets to get the same simulation results. It has great significance for enhancing the effectiveness of NRCS and SAR image simulation. This model exhibits robustness and efficiency in SAR imaging of ocean surfaces and ship wakes. It is demonstrated to be reasonable compared to advanced analytical models and experimental data. In this paper, we focus more on evaluating the scattered field amplitude rather than precisely treating its phase, and this work will be considered in a future investigation.

## Figures and Tables

**Table 1:** The values of parameters in simulation.

Parameter	Value
Carrier frequency *f* (GHz)	14
Pulsed duration 𝜏 (us)	2
Bandwidth ∆f (MHz)	200
Radar velocity *v* (m/s)	300
Azimuth antenna dimension *L* (m)	3

## Data Availability

Not applicable.
